# P83 Phage–antibiotic synergy to combat carbapenemase producing strains of *Enterobacter cloacae* complex for biofilm-related infections

**DOI:** 10.1093/jacamr/dlaf230.090

**Published:** 2025-12-04

**Authors:** Kash Haq, Martin Figgitt, David Lee

**Affiliations:** Birmingham City University, Birmingham, UK; Birmingham City University, Birmingham, UK; Birmingham City University, Birmingham, UK

## Abstract

**Background:**

Infected wounds and chronic infections have become a global issue and aetiology is complicated by the increase of antibiotic resistance in bacteria. The Gram-negative bacteria *Enterobacter cloacae* complex has become an emerging threat and are routinely associated with non-healing wounds and biofilm related infections. Phage therapy has been used for more than a century to treat bacterial infections that are caused by MDR organisms.

**Methods:**

This study investigated the efficacy of a cocktail of two lytic bacteriophages (vB_EluM_RZH and vB_EholP_HHH) isolated from the River Chelt and the antibiotic meropenem in inhibiting and degrading the biofilms produced by certain strains of MDR Enterobacteriaceae expressing carbapenemases. Phages underwent physical and genomic characterization, then validated by the NCBI. Biofilm metabolic activity and biomass was measured in a modified chequerboard assay using MBEC format. Phage–antibiotic synergy was calculated and harmonized using synergy scoring models HSA, Loewe, Bliss and Zip. The influence of phages on human fibroblasts cells was evaluated through cell metabolic activity and the release of lactate dehydrogenase (LDH).

**Results:**

Both phages showed broad-host range, producing bacteriolytic activities against strains of *E. cloacae* complex and *Escherichia coli*. They showed short replication times, large burst sizes with rapid adsorptions. They exhibited significant stability under various environmental conditions. Genomic analysis revealed that both phages are genetically distinct phages, belonging to the Tesseptimavirus and Karamivrus genus, with the lack of toxin, virulence, lysogeny and antibiotic resistance genes. Checkboard assay results indicated phage and antibiotic synergy (PAS) against 24 h and 48 h biofilms when compared to the control or a single agent alone . Synergy scores from all models indicated a top score of 30 (combination of antibiotic at 128–256 μg/ml and phage cocktail 6–7 PFU/ml) demonstrated the best results. Traditional fractional inhibitory concentration (FIC) calculations also indicated a synergy effect (≤0.5). When human fibroblasts were treated with the phages, no differences in LDH levels or metabolic activity were observed when compared to the untreated control (*P*<0.001). Furthermore, phages and phage–antibiotic combinations could significantly reduce the bacterial load (cfu/mL) when targeted against infected fibroblasts, without causing damage to the dermal cells, however, antibiotic alone did not significantly reduce the bacterial load and indicated a slight cytotoxic effect on the cells.

**Conclusions:**

These findings suggest bacteriophage cocktail combined with meropenem improved the inhibition and destruction of biofilms when compared to testing the agents alone, moreover the combination demonstrated a positive outcome when tested with fibroblasts. These results contribute to the expanding field of biofilm dynamics and the potential of bacteriophages as a novel treatment strategy

